# Exploring the Protective Effects of Taxifolin in Cardiovascular Health: A Comprehensive Review

**DOI:** 10.3390/ijms26168051

**Published:** 2025-08-20

**Authors:** Hwan-Hee Sim, Ju-Young Ko, Dal-Seong Gong, Dong-Wook Kim, Jung Jin Kim, Han-Kyu Lim, Hyun Jung Kim, Min-Ho Oak

**Affiliations:** 1 Department of Pharmacy, College of Pharmacy, Mokpo National University, Muan 58554, Republic of Korea; qqwwee114@naver.com (H.-H.S.); herolegend@hanmail.net (J.-Y.K.); nh4011@gmail.com (D.-S.G.); 2 Convergence Center for Green Anti-Aging Research, Muan 58554, Republic of Korea; wekkiri@naver.com (J.J.K.); limhk@mokpo.ac.kr (H.-K.L.); 3 Department of Pharmaceutical Engineering, College of Food & Pharmaceutical Engineering, Mokpo National University, Muan 58554, Republic of Korea; dbkim@mokpo.ac.kr; 4 Department of Marine and Fisheries Resources, Mokpo National University, Muan 58554, Republic of Korea

**Keywords:** taxifolin, cardiovascular health, SGLT2

## Abstract

Taxifolin is a natural flavonoid found in a variety of plants, including Siberian larch (*Larix sibirica*) and milk thistle (*Silybum marianum*), that has attracted attention for its multifaceted pharmacological properties, including cardioprotective effects. Through its antioxidant and anti-inflammatory activities, taxifolin has shown significant therapeutic potential in cardiovascular diseases such as atherosclerosis, myocardial ischemia, and diabetic cardiomyopathy. This review highlights the cardioprotective effects of taxifolin in preclinical models of atherosclerosis, ischemia/reperfusion injury, and diabetic cardiomyopathy. Taxifolin contributes to its cardioprotective effects through key mechanisms such as modulation of pathways such as PI3K/AKT and JAK2/STAT3, inhibition of NADPH oxidase, and modulation of nitric oxide production. Recent studies have shown that taxifolin can affect glucose metabolism by modulating sodium–glucose transporter (SGLT) expression, potentially enhancing the cardioprotective effects of SGLT2 inhibitors. Given the emerging role of SGLT2 inhibitors in the management of cardiovascular disease, further investigation of the interaction of this pathway with taxifolin may provide new therapeutic insights. Although taxifolin has multifaceted potential in the prevention and treatment of cardiovascular disease, further studies are needed to better understand its mechanisms and validate its efficacy in different disease stages. This review aims to provide a rationale for the clinical application of taxifolin-based cardiovascular therapies and suggest directions for future research.

## 1. Introduction

Cardiovascular disease (CVD) is one of the leading causes of death worldwide and manifests itself in various forms, including hypertension, atherosclerosis, and myocardial infarction [1]. These diseases are mainly caused by multiple pathological mechanisms, including oxidative stress, inflammatory response, and dyslipidemia, and there is an ongoing need to develop effective therapeutics to modulate these mechanisms [2,3]. Recently, natural substances from the flavonoid family have gained attention in the prevention and management of CVDs, and taxifolin has emerged as a promising candidate for its efficacy and safety [4]. Taxifolin is a flavonoid primarily derived from plants that exhibits potent antioxidant and anti-inflammatory activities, inhibiting the production of reactive oxygen species and inflammatory cytokines, which are major contributors to CVD [5,6]. In addition, taxifolin plays an important role in maintaining vascular health by protecting vascular endothelial cell function and regulating cholesterol metabolism [7]. These properties suggest that taxifolin may have preventive or therapeutic potential in a variety of CVDs, including hypertension, atherosclerosis, and hyperlipidemia.

Focusing on the protective effects of taxifolin on the cardiovascular system, this review synthesizes recent studies to explore its mechanism of action and clinical potential. In doing so, this review aims to further analyze the potential role of taxifolin in the prevention and treatment of CVDs and provide directions for future research and clinical applications.

## 2. Taxifolin

**Figure 1:**
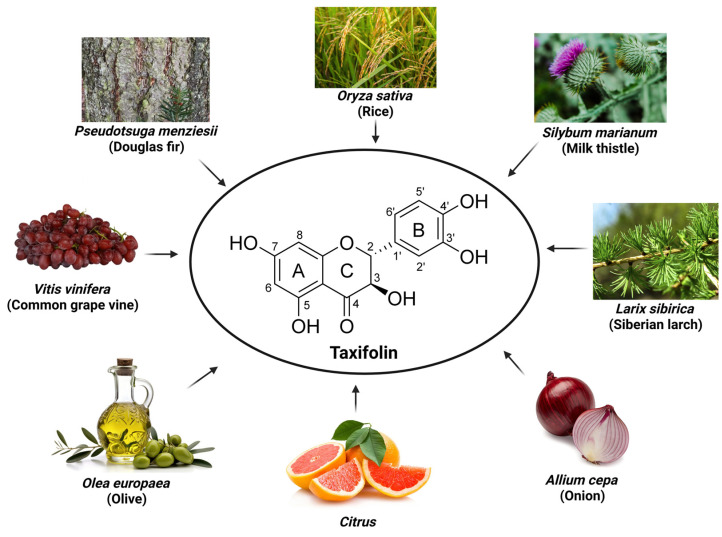
Taxifolin origin and structure.

Originally isolated from the bark of Douglas pine wood (*Pseudotsuga menziesii*, Pinaceae) [8], taxifolin, also known as dihydroquercetin or 3,5,7,3′,4′-pentahydroxyflavanone, has since been found in a variety of plants. It is widely distributed in nature, including Siberian larch (*Larix sibirica*, Pinaceae), milk thistle (*Silybum marianum*, Asteraceae), and onions (*Allium cepa*, Amaryllidaceae), and is recognized as an important bioactive substance [9,10] (Figure 1).

Milk thistle and onion, in particular, have been recognized for their beneficial effects on cardiovascular health, further highlighting the therapeutic potential of taxifolin [11,12,13]. In addition, taxifolin exhibits a variety of pharmacological activities, including antioxidant, anti-inflammatory, anticancer, and neuroprotective properties. Due to these pharmacological activities, it is widely studied in various therapeutic applications such as cancer, CVD, liver disease, and as a food additive. It is also used as an ingredient in dietary supplements for its health-promoting properties [14].

Taxifolin (C_15_H_12_O_7_, molar mass 304.25 g/mol) belongs to flavanonol (2,3-dihydroflavonol), a subclass of flavonoids [15]. The structure of taxifolin consisting of two phenyl groups (A and B) bonded to O-heterocycle C, which is a key factor in determining the chemical and biological properties of taxifolin [16]. Specifically, the A ring of taxifolin contains hydroxyl groups bonded at positions C-5 and C-7, while hydroxyl groups at the C-3′ and C-4′ positions of the B ring contribute to mitigating oxidative stress by neutralizing free radicals and stabilizing electron distribution [17,18]. The C ring has a hydroxyl group at position C-3 and a carbonyl group at position C-4, which plays an important role in chelating metal ions [19]. The stereoisomeric structure of taxifolin is characterized by the two chiral centers (C-2 and C-3) in the C ring. It was known that the dihydroflavonols with *trans*-diaxial H-2/H-3 position are more stable than the *cis*-compounds [18], and the absolute structure of a major isomeric compound is (2*R*,3*R*)-(+)-taxifolin [20,21] (Figure 1). The multiple hydroxyl groups in taxifolin exert potent antioxidant activity by increasing its high solubility in water and ability to form hydrogen bonds. In addition, the carbonyl group in the C ring and the dihydroxyl group in the B ring are involved in metal ion chelation, which inhibits reactive oxygen species (ROS) generation and reduces metal-catalyzed oxidation reactions, thus preventing oxidative damage [19]. The unique arrangement of hydroxyl and carbonyl groups shows that it exhibits antioxidant and anti-inflammatory effects, making it a promising compound for therapeutic use [22]. Research into the structure–activity relationship of taxifolin continues to reveal its potential to combat oxidative stress and inflammation, highlighting its importance in the realm of natural antioxidants.

## 3. Pharmacological Activity of Taxifolin

**Figure 2:**
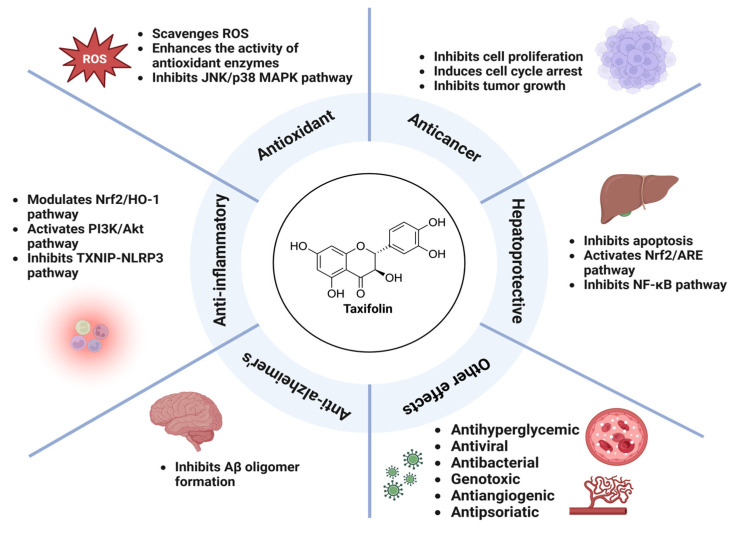
Schematic representation of the pharmacological effects and mechanism of action of taxifolin.

Taxifolin exhibits a variety of pharmacological effects, including antioxidant, anti-inflammatory, hepatoprotective, anticancer, and neuroprotective (Figure 2).

It effectively scavenges ROS, enhances antioxidant enzymes, and inhibits inflammatory responses through the NF-κB and PI3K/Akt pathways. Taxifolin protects the liver by reducing oxidative stress and apoptosis, while also demonstrating anticancer effects through cell cycle arrest and tumor suppression [23]. In Alzheimer’s disease models, it attenuates amyloid beta accumulation and neuroinflammation. Taxifolin also exhibits antibacterial, antiviral, and antiangiogenic effects, showing broad therapeutic potential.

### 3.1. Antioxidant Activity

Flavonoids exhibit potent antioxidant activity in the body as free radical scavengers and complexing agents for metal ions [24,25]. Among flavonoids, taxifolin is known to have particularly potent antioxidant activity [26]. This compound effectively scavenges ROS to reduce oxidative stress, which prevents cell damage and plays an important role in the prevention of a variety of chronic diseases. Studies have shown that taxifolin has greater antioxidant capacity than common flavonoids and is more effective than quercetin, the leading antioxidant. Taxifolin inhibited lipid peroxidation by lowering malondialdehyde levels in a hepatitis model, and was 3.4 times more effective than quercetin at a dose of 100 mg/kg and 4.9 times more effective at a dose of 300 mg/kg [27]. Studies have shown that taxifolin has a potent antioxidant effect in a CCl_4_-induced hepatitis model [28]. The study showed that taxifolin enhances the activity of antioxidant enzymes such as superoxide dismutase (SOD) and catalase, which are important for neutralizing ROS. Another study also demonstrated the antioxidant efficacy of taxifolin to scavenge ROS and prevent cell death in bmMSCs (bone marrow-derived mesenchymal stem cells) damaged by hydroxyl radicals by inhibiting the JNK/p38 MAPK signaling pathway [29]. The ability to chelate iron ions has also been identified as one of the antioxidant effects of taxifolin [19]. It was found that taxifolin inhibits the production of ROS by binding to iron ions and exerts its antioxidant activity through its ability to chelate iron ions.

### 3.2. Anti-Inflammatory Activity

Protection against liver injury is one of taxifolin’s notable benefits, achieved primarily through the inhibition of oxidative stress and inflammatory responses. Studies have shown that taxifolin mitigates cisplatin-induced nephrotoxicity by modulating the Nrf2/HO-1 pathway, an important regulator of oxidative stress and inflammation [30]. Nrf2 is a key defense mechanism against oxidative stress by promoting the expression of antioxidant enzymes within cells. Taxifolin activates Nrf2 to induce the expression of HO-1, which plays a role in suppressing oxidative stress and inflammation. This regulation leads to a reduction in pro-inflammatory cytokines such as TNF-α and IL-6 and decreases markers of oxidative stress. By inhibiting these inflammatory mediators, taxifolin protects kidney tissue. Taxifolin has also been shown to modulate inflammatory responses through the PI3K/Akt signaling pathway [31]. In a rat model with metabolic syndrome, taxifolin ameliorated impairments in glucose metabolism and water–salt metabolism by activating the PI3K/Akt pathway. Notably, modulating this pathway reduced the production of pro-inflammatory cytokines, effectively suppressing inflammatory responses in kidney tissue. In another study, taxifolin was shown to play an important role in suppressing inflammatory responses in high glucose-stimulated rat microglia [32]. The study demonstrated that taxifolin attenuates inflammatory responses by modulating the TXNIP-NLRP3 axis. In a high-glucose environment, TXNIP expression increased, which activated NLRP3 inflammasomes, leading to the release of inflammatory cytokines. However, taxifolin effectively suppressed the excessive inflammatory response in microglia by inhibiting TXNIP expression and reducing the activation of NLRP3 inflammasomes. This reduced the production of inflammatory mediators and prevented neuronal damage. Taxifolin prevented various tissue damage by inhibiting the production of inflammatory cytokines and alleviating oxidative stress and inflammatory responses.

### 3.3. Hepatoprotective Activity

Taxifolin protects against liver injury through its primary mechanism of inhibiting oxidative stress and inflammatory responses. Studies have shown that taxifolin attenuates liver injury in a mouse model of CCl_4_-induced acute liver injury by reducing oxidative stress through activation of antioxidant enzymes and inhibiting inflammatory responses in hepatocytes [33]. Specifically, taxifolin maintained glutathione levels, reduced the accumulation of malondialdehyde, and prevented damage to cell membranes. In addition, a study was published showing that taxifolin enhances the antioxidant defense system of hepatocytes and inhibits hepatocyte apoptosis by activating the Nrf2/ARE pathway in an acetaminophen-induced acute liver injury model [34]. In this study, taxifolin reduced ROS in liver cells and inhibited apoptosis by regulating the Bax/Bcl-2 ratio. Taxifolin was found to suppress inflammatory responses and promote cell survival in acute alcohol-induced liver injury by regulating the NF-κB pathway and PI3K/Akt pathway. Taxifolin attenuated liver inflammation by decreasing the expression of pro-inflammatory cytokines and increasing the expression of anti-inflammatory cytokines. The protective effects of taxifolin were evaluated in mice with induced hepatic encephalopathy and found that it contributed to alleviating the symptoms of hepatic encephalopathy by suppressing inflammation and oxidative stress in the liver and brain [35]. The study confirmed that taxifolin alleviated hepatic encephalopathy by maintaining the integrity of the blood–brain barrier and reducing neuroinflammation. These studies demonstrate the potential of taxifolin to protect the liver through its antioxidant, anti-inflammatory, and cytoprotective effects in various liver injury models.

### 3.4. Anticancer Activity

As a flavonoid, taxifolin has gained significant attention in cancer research for its potent anticancer properties, primarily through interactions with key signaling pathways and regulation of the cell cycle. Studies have shown that treatment of various cancer cell lines, including HeLa (human cervical cancer), HepG2 (human liver cancer), and MDA-MB-231 (human breast cancer), with taxifolin at concentrations ranging from 10 μM to 100 μM, exhibits anticancer effects [36]. The study demonstrated that taxifolin inhibits cancer cell proliferation in a dose-dependent manner by inducing cell cycle arrest, mainly in the G_2_/M phase. Cell cycle arrest is mediated by the regulation of important cell cycle regulators such as cyclins and cyclin-dependent kinase; specifically, taxifolin inhibits these regulators to prevent the transition from G_2_ phase to M phase, effectively halting cell cycle progression and thus inhibiting cancer cell proliferation. The study also examined the effects of taxifolin on the Wnt/β-catenin signaling pathway, a pathway that is often dysregulated in cancer. Taxifolin was shown to inhibit tumor growth by enhancing the nuclear translocation of β-catenin, while inducing the activation of Wnt target genes that promote cell death pathways. This cell cycle inhibition and activation of cell death signaling contributed to a significant reduction in tumor growth in the models used in the study. A study in SKH-1 hairless mice demonstrated that topical application of taxifolin significantly reduced the development and proliferation of UVB-induced skin tumors [37]. By inhibiting the phosphorylation of EGFR, taxifolin effectively downregulated the subsequent activation of the PI3K/Akt signaling pathway, which is known to promote cell survival, proliferation, and tumor growth.

### 3.5. Anti-Alzheimer’s Activity

Alzheimer’s disease (AD) is a neurodegenerative disease characterized by the gradual death of brain nerve cells due to the buildup of abnormal proteins, such as amyloid-β (Aβ) protein, in the brain. Taxifolin has attracted attention for its neuroprotective effects related to AD, and several studies have demonstrated its promising anti-AD effects. Studies have shown that taxifolin is effective in inhibiting Aβ oligomer formation, restoring vascular structural integrity, and improving memory function in an Aβ-injected rat model [38]. This study highlighted the mechanisms by which taxifolin improves vascular permeability and restores cognitive function in cerebral amyloid angiopathy. In addition, the effectiveness of taxifolin in preventing synapse formation impairment and memory deficits in the H9C2 cardiomyocyte model induced by β-amyloid was also demonstrated [39]. Taxifolin reduced inflammatory responses by inhibiting the cPLA**_2_**/PGE**_2_** pathway, resulting in neuroprotection and improved cognitive function. Studies have also shown that taxifolin exhibited multifaceted neuroprotective effects in an Aβ-induced mouse model, enhancing neuronal survival and reducing damage through the PI3K/Akt pathway [40]. In this study, taxifolin significantly reduced Aβ protein accumulation, alleviated vascular damage through improved vascular permeability and normalization of vascular structure, and restored the function of vascular endothelial cells. In addition, taxifolin reduced neuronal death by activating the PI3K/Akt pathway and regulated neuroinflammatory responses by inhibiting the NF-κB pathway. It was also demonstrated that the combined treatment of taxifolin and cilostazol reduced Aβ accumulation and neurotoxicity by inhibiting the p-JAK2/p-STAT3/NF-κB/BACE1 signaling pathway [41].

### 3.6. Other Pharmacological Activities

In addition to its antihyperglycemic effects, which improve insulin sensitivity and lower blood glucose levels [42,43,44], taxifolin has demonstrated a variety of pharmacologic actions. These include antiviral [45], antibacterial [46,47], and genotoxic activities [48], as well as antiangiogenic activity that inhibits abnormal blood vessel formation [49]. Anti-psoriatic effects have also been shown, providing potential benefits in psoriasis management by modulating additional inflammatory pathways [50].

## 4. Taxifolin and the Cardiovascular System

**Figure 3:**
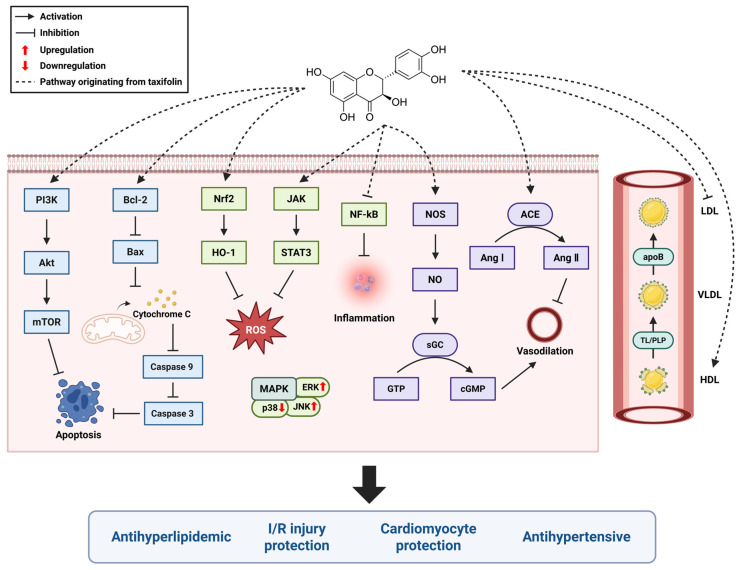
Proposed molecular mechanisms of taxifolin in cardiovascular protection.

Research on taxifolin has explored its potential benefits in cardiovascular health, liver protection, cancer prevention, and other areas. Various experimental and epidemiologic studies have shown that consumption of flavonoid-rich foods is associated with a reduced risk of CVD [51]. Taxifolin has been recognized for its antioxidant, anti-inflammatory, and antihyperlipidemic properties, which may play an important role in the prevention and treatment of CVD, including antihypertensive effects and cardiomyocyte protection [52,53]. Recently, various mechanisms of action have been investigated, showing promising results in blood pressure regulation, cholesterol metabolism, and protection against myocardial ischemia/reperfusion injury (Table 1 and Figure 3). Therefore, taxifolin is expected to contribute to the alleviation of CVDs such as atherosclerosis, hypertension, and myocardial infarction.

### 4.1. Antihypertensive

Taxifolin has shown antihypertensive activity in experimental models of hypertension. Studies have shown that taxifolin lowers blood pressure by enhancing vasorelaxant function and inhibiting the activity of inflammatory cytokines in the blood vessels [59]. Endothelial function was significantly improved in the taxifolin-treated group, with an increased vasorelaxant response to acetylcholine and a decreased phenylephrine-induced vasoconstrictor response. Taxifolin also inhibited vessel wall thickening, reduced damage to endothelial cells, and induced structural improvements in blood vessels. Studies have also demonstrated the antihypertensive effects of taxifolin in hypertension models [62]. In SHR and IHR models, taxifolin effectively lowered blood pressure, and its mechanism of action was shown to be through the improvement of vasorelaxant responses and the alleviation of oxidative stress and inflammatory responses. Another study used a radiation-induced hypertension model and found that taxifolin effectively suppressed hypertension by inhibiting angiotensin-converting enzyme (ACE) activity and preventing excessive production of angiotensin II, which causes vasoconstriction [61]. By inhibiting ACE activity and preventing the overproduction of angiotensin II, which causes vasoconstriction and elevated blood pressure, taxifolin shows promise as a treatment for hypertension. In a rat model of aging and increased oxidative stress and inflammatory response, taxifolin also inhibited ACE activity [58]. Taxifolin demonstrated antihypertensive effects by lowering blood pressure in elderly hypertensive rat [60]. Taxifolin treatment significantly lowered systolic blood pressure through mechanisms related to the modulation of the endothelial function and reduction in oxidative stress. Specifically, taxifolin increases the bioavailability of nitric oxide (NO), which promotes vasodilation and reduces vascular resistance. This demonstrates the therapeutic potential of taxifolin in the management of hypertension in the elderly population, who are vulnerable to oxidative stress and vascular dysfunction.

### 4.2. Cardiomyocyte Protection

In various cardiac injury models, protective effects on cardiomyocytes have been demonstrated by taxifolin. Studies have demonstrated that taxifolin attenuates oxidative stress and cardiac structural damage in an acrylamide-induced cardiac injury model [70]. It has also demonstrated protective effects against isoproterenol-induced cardiac injury [6]. Taxifolin exerted its cardioprotective effects by regulating oxidative stress and inflammatory responses by activating the Nrf2/HO-1 pathway. Additionally, taxifolin has been found to prevent cardiac hypertrophy and fibrosis while improving cardiac function under pressure overload via the ERK1/2, JNK1/2, and Smad signaling pathways [52]. Furthermore, taxifolin was effective in preventing and alleviating cardiac damage in diabetic cardiomyopathy by activating the JAK2/STAT3 pathway, suppressing oxidative stress, and inhibiting apoptosis through decreased caspase-3 expression and increased Bcl-2 expression [72]. Diabetic cardiomyopathy, a complication of diabetes that causes structural and functional damage to the heart, was significantly alleviated by taxifolin treatment.

### 4.3. Myocardial Ischemia/Reperfusion (I/R) Injury Protection

Several studies have demonstrated the cardioprotective effects of taxifolin against ischemia/reperfusion injury via modulation of oxidative stress and apoptotic pathways. Taxifolin exerted myocardial protective effects by inhibiting oxidative stress and endoplasmic reticulum stress-induced apoptosis through activation of the PI3K/Akt signaling pathway, resulting in a significant reduction in cardiac tissue damage [73]. In addition, taxifolin reduced apoptosis by preventing the loss of mitochondrial membrane potential, inhibiting the release of cytochrome C, and inhibiting the activation of caspase-9 and caspase-3 [5]. Taxifolin alleviated oxidative stress and apoptosis by reducing ROS production, increasing the expression of Bcl-2, and inhibiting the expression of the pro-apoptotic protein Bax. In addition, blood levels of LDH and CK-MB were significantly reduced in the taxifolin-treated group, confirming that myocardial injury was inhibited. Morphological analysis of myocardial tissue showed that taxifolin preserved the myocardial structure by reducing myofibrillar destruction, edema, and inflammatory cell infiltration caused by I/R injury.

### 4.4. Antihyperlipidemic

Hyperlipidemia is an abnormally high level of triglycerides and low-density lipoprotein (LDL) cholesterol in the blood, which puts a strain on the cardiovascular system and increases the risk of conditions like atherosclerosis and heart attack. Taxifolin has been shown to be effective in lowering serum cholesterol and LDL cholesterol levels in several studies. Taxifolin has been shown to be effective in lowering serum cholesterol and LDL cholesterol levels in several studies. Studies have shown that taxifolin lowers serum cholesterol levels by lowering LDL cholesterol and improving high-density lipoprotein (HDL) cholesterol balance [55]. In addition, the antioxidant properties of taxifolin reduce oxidative stress in lipids, which not only prevents oxidation of LDL but also benefits overall cardiovascular health. In addition to this, taxifolin reduces oxidative stress and prevents oxidative damage associated with fat accumulation in the liver. Administration of taxifolin-rich tea to rats increased the activity of the antioxidant enzymes SOD and catalase, which contributed to the reduction in cellular damage caused by oxidative stress [54]. In another study, taxifolin was shown to reduce the production and secretion of LDL and very low-density lipoprotein (VLDL) in the liver [57]. This was achieved by inhibiting apoptosis and secretion of apoB and increases secretion of apoA-I, suggesting that taxifolin may be effective in the management of hyperlipidemia.

### 4.5. Clinical Studies

Various preclinical studies have recognized the cardioprotective effects of taxifolin, including improving cardiovascular health through antioxidant and anti-inflammatory actions, preventing cardiac injury, and enhancing vascular endothelial function. According to the recent systematic review for natural and semi-synthetic flavonoid drugs, a total of 19 flavonoid-based drugs have been approved for medical prescription, and 30% of them are used for the treatment of cardiovascular diseases [74]. However, despite these positive results, to date, few clinical studies have been conducted on the effects of taxifolin on CVD. There is also a lack of data on the safety of taxifolin, appropriate dosage, and possible side effects of long-term use. Therefore, clinical studies in patients with CVD will be an important step in developing taxifolin into a real treatment. Future studies should systematically validate the cardioprotective effects of taxifolin in different patient populations, evaluate its combination with other cardiovascular drugs, and assess its long-term efficacy. This will play an important role in broadening the clinical applicability of taxifolin and establishing its safety.

## 5. Future Research Directions

**Figure 4:**
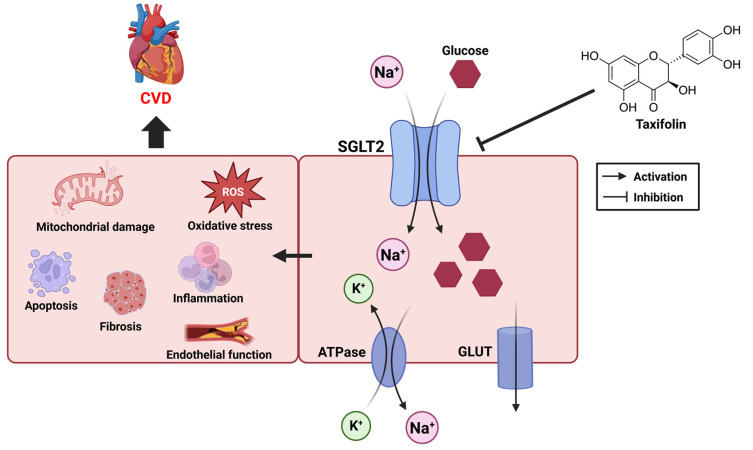
Pathological effects of SGLT2 activity on cardiovascular health and potential modulation by taxifolin.

Sodium–glucose transporter (SGLT) and cardiovascular health have been the focus of recent research. The SGLT is an important protein that regulates the intracellular transport of glucose and has two main forms, SGLT1 and SGLT2. SGLT1 is primarily responsible for the uptake of glucose and galactose in the small intestine, while SGLT2 is responsible for the reabsorption of glucose in the kidneys [75]. These transporters utilize sodium concentration gradients to efficiently move glucose into cells, which is essential for the regulation of glucose metabolism in the body [76] (Figure 4).

SGLT2 inhibitors work primarily to lower blood sugar by blocking the reabsorption of glucose in the kidneys. By inhibiting SGLT2′s ability to move sodium ions and glucose together into cells, glucose is excreted in the urine [77]. This reduces the concentration of glucose in the body and improves insulin secretion, which is effective in diabetes management [78]. However, SGLT2 inhibitors do more than just control blood sugar; they have also been shown to have a positive impact on cardiovascular health [79]. In addition to glycemic control, recent studies have shown that SGLT2 inhibitors provide significant cardiovascular benefits, including reduced risk of heart failure and improved endothelial function [80,81]. Given the known cardioprotective properties of taxifolin, studies elucidating its effects on these mechanisms may contribute to the development of new therapeutic strategies. In future studies, it will be important to further explore the mechanisms of its potential interaction with SGLT2 and its inhibitors.

### 5.1. CVD and Sodium–Glucose Transporter

Under normal conditions, glucose is an essential energy source for heart contraction, but in hyperglycemia and metabolic syndrome, it can have negative effects on the cardiovascular system, including endothelial dysfunction, increased inflammation, and oxidative stress. SGLT2 inhibitors reduce inflammatory responses, decrease oxidative stress, and improve endothelial function. They also exert cardioprotective effects by reducing the burden on the heart through fluid regulation [82,83,84] (Figure 4). SGLT2 inhibitors inhibit atherosclerosis by normalizing the expression of inflammation-related genes such as TNF-α, IL-6, ICAM-1, MMP2, and MMP9 [85]. These drugs reduce oxidative stress by reducing ROS production and improve cardiac function by preventing cellular damage [86]. They have also demonstrated therapeutic effects on endothelial dysfunction by reducing plasma levels of CAM, a marker of endothelial dysfunction [87]. Additionally, SGLT2 inhibitors promote the excretion of sodium from the kidneys, which reduces water in the body, which in turn lowers the pressure on the heart, helping to alleviate symptoms in patients with heart failure [88]. Through these mechanisms, SGLT2 inhibitors play an important role in promoting cardiovascular health.

Several clinical studies have confirmed the cardioprotective effects of SGLT2 inhibitors. In the EMPA-REG OUTCOME study, empagliflozin was shown to reduce the risk of cardiovascular death by 38% and hospitalization for heart failure by 35% in patients with type 2 diabetes [89]. The CANVAS program also found that canagliflozin reduced the risk of cardiovascular death by 20% and reduced the incidence of cardiovascular events by 14% [90]. In the DECLARE-TIMI 58 study, dapagliflozin was found to be effective in reducing heart failure hospitalization rates by 27% [91]. Similar results were confirmed by ertugliflozin in the VERTIS CV study, which found that while the drug did not significantly reduce the direct occurrence of cardiovascular events, it played a role in reducing heart failure-related hospitalizations [92]. These studies suggest that SGLT2 inhibitors may be important therapeutic agents in the prevention and management of CVD, not just in the management of diabetes. These mechanisms have important implications for the prevention of cardiovascular and renal disease, especially in diabetic patients. However, research involving SGLTs is still in its infancy, and future studies are needed. It is important to elucidate how SGLT inhibitors interact with different metabolic pathways and the mechanisms by which they contribute to the prevention of CVD. In addition, research is needed on the effects of natural substances such as taxifolin on the function of SGLT and their interactions with SGLT inhibitors. This may contribute to the development of new therapeutic strategies. These research directions will contribute to a deeper understanding of the physiological functions of SGLTs and clarify their role in the prevention and treatment of CVDs. By exploring the various functions of SGLTs and their association with the cardiovascular system, new insights into the prevention and treatment of related diseases may be gained.

### 5.2. SGLT and Taxifolin

Research has shown that taxifolin plays an important role in ameliorating glucose and water–salt metabolic disturbances in metabolic syndrome rats [31]. The study found that taxifolin exerts these effects through the PI3K/Akt signaling pathway, suggesting that taxifolin influences metabolic regulation. Specifically, taxifolin reduces the expression of SGLT2 and GLUT2, which prevents excessive reabsorption of glucose, promotes the excretion of glucose, and contributes to glycemic control. This suggests that taxifolin may act in a similar manner to SGLT inhibitors to help regulate blood sugar. In addition, by lowering SGLT2 levels and reducing proximal glucose reabsorption, normal tubuloglomerular feedback mechanisms are restored, which may contribute to reduced glomerular filtration. Another study examined the combined effect of taxifolin and dapagliflozin and found that it lowered intrarenal blood pressure and improved blood flow [93]. Dapagliflozin is an SGLT2 inhibitor that blocks the reabsorption of glucose and sodium in the kidneys, reducing the body’s inflammatory response and promoting sodium and water excretion. In this study, the combination of taxifolin and dapagliflozin was more effective than monotherapy in improving colistin-induced nephrotoxicity. The combination of taxifolin and dapagliflozin effectively alleviated colistin-induced nephrotoxicity by inhibiting inflammatory cytokine expression in kidney tissue, increasing antioxidant enzyme activity, significantly reducing kidney damage, and lowering blood creatinine and urea nitrogen levels, which are markers of kidney function. These mechanisms suggest that the synergistic effects of the two compounds may play an important role in kidney protection. Further studies are needed to better understand the interaction of SGLT2 inhibitors and taxifolin, especially their effects on CVD. Such studies may clarify the effects of taxifolin on SGLTs and provide new approaches to the treatment of CVD.

### 5.3. Limitations

This review summarizes preclinical evidence on taxifolin’s cardioprotective potential, but several limitations should be noted. Most data derive from in vitro and animal models, with limited human clinical evidence, making translational relevance uncertain. Long-term safety, pharmacokinetics, and drug–drug interactions with standard cardiovascular therapies have not been systematically assessed. Although short-term studies suggest a favorable profile, rigorous evaluations of chronic and organ-specific toxicity are lacking. Marked heterogeneity in dosing regimens and treatment durations across preclinical studies further complicates extrapolation to humans and hinders determination of optimal dosing and exposure. Finally, this work was conducted as a narrative review rather than a systematic review under PRISMA 2020 guidelines. As such, the possibility of incomplete study capture, selection bias, or interpretive bias cannot be excluded, and the transparency and reproducibility of study selection may be limited. While a comprehensive PRISMA-based systematic review may be premature at this stage due to the paucity of clinical data, such an approach will become essential once more robust evidence emerges. Future systematic reviews will be necessary to rigorously evaluate taxifolin’s efficacy, safety, and its comparative and combination effects with other flavonoids and pharmacotherapies.

## 6. Conclusions

Taxifolin is a natural flavonoid with great potential for improving cardiovascular health through its antioxidant, anti-inflammatory, and vascular protective effects, and various studies have demonstrated its potential in the prevention and treatment of cardiovascular disease. In particular, taxifolin plays an important role in reducing blood pressure and mitigating cardiac injury and vascular structural damage through a variety of pharmacological effects. The preclinical studies presented in this review have demonstrated that taxifolin has cardioprotective effects, including blood pressure reduction, myocardial protection, and prevention of ischemic injury, and that these effects are exerted primarily through ROS reduction, anti-inflammatory mechanisms, and modulation of various cellular pathways.

Nevertheless, clinical studies of taxifolin in the prevention and treatment of cardiovascular disease are currently limited, and there is a lack of data on its long-term safety and efficacy in various patient populations. Therefore, it will be important for future studies to clinically validate the effectiveness of taxifolin, evaluate its potential for combination with other cardiovascular therapies, and determine the optimal dose and method of administration. These studies are expected to establish taxifolin as a promising therapeutic agent for cardiovascular healthcare.

## Figures and Tables

**Table 1:** Taxifolin research in cardiovascular disease.

Pharmacological Activities	Taxifolin Dose	Molecular Targets/ Mechanisms	Study Type		Ref.
			In Vitro	In Vivo	
Antihyperlipidemic	0.05% (*w*/*w*)	Liver total cholesterol ↓ Liver phospholipid ↓ TBARS ↓		Wistar-strain rat	[54]
	0.05% (*w*/*w*) 0.1% (*w*/*w*)	Total cholesterol ↓ HDL-cholesterol ↑		Wistar-strain rat	[55]
	2 μM	NO2 radical scavenging MPO/Nitrite-induced LDL oxidation ↓	Human LDL		[56]
	50, 100, 200 μM	HMG-CoA reductase ↓ apoB ↓ apoA-I ↑	HepG2 cell		[57]
Antihypertension	30, 100 μg/kg	ACE activity ↓ ROS/RNS ↓ 5-lipoxygenase ↓ NADPH oxidase ↓		L-NAME and dexamethasone- treated Wistar rat	[58]
	20 mg/kg	Blood pressure ↓ COX2 ↓ Total NOS activity ↑ IL-10 ↑		SHR	[59]
	100 μg/kg	Systolic blood pressure ↓		Aged Wistar rat	[60]
	100 μg/kg	ROS scavenging ACE activity ↓		X-ray-irradiated Wistar rat	[61]
	20 mg/kg	Blood viscosity ↓ Vasodilation ↑		SHR	[62]
	1–300 μM	eNOS ↑ PI3K/eNOS/cGMP pathway	Porcine coronary artery rings and endothelial cell		[63]
Cardiomyocyte protection	30, 50 μM	Oxidative stress ↓ Cell apoptosis ↓ Cell adhesion ↓	HUVECs THP-1 cell		[64]
	10 μM 20 mg/kg	Ferroptosis ↓ Oxidative stress ↓ miR-200a expression ↑ Nrf2 ↑	H9C2 cell	Dox-treated C57BL/6 mice	[65]
	20, 50 mg/kg	Malondialdehyde ↓ Oxidative stress ↓ Nrf2, HO-1 ↑		ISO-treated Swiss albino mice	[6]
	200 μM	iNOS, OPN ↓ HMGB1 ↑	H9C2 cell		[66]
	0.5 μM	Cell apoptosis ↓ Oxidative stress ↓ Cytochrome P450 ↑	Primary chicken cardiomyocytes		[67]
	0.5 μM	Ca^2+^ overload ↓ CaMKII-RIPK3 ↓	Primary chicken cardiomyocytes		[68]
Cardioprotection	0.5 μM	IL-6/JAK/STAT3 ↓ PPARs/PGC-1α ↑	Primary chicken cardiomyocytes		[69]
	50 mg/kg	Malondialdehyde ↓ Troponin I ↓ ROS scavenging Inhibition of NF-kB pathway		Acrylamide-treated Wistar rat	[70]
	100 μg/mL 200 mg/kg	HIF-1α ↓ eNOS, VEGF-α, TGF-β, FGF21 ↑ Activation of PI3K/AKT/mTOR/STAT3 pathway	H9C2 cell		[71]
	5, 10, 25, 50 μM 0.2% (*w*/*w*)	Malondialdehyde ↓ MMP-9, TIMP1 ↓ Inhibition of MAPKs, TGF-β/Smad pathway	Neonatal rat cardiomyocytes	TAC model C57BL/6 mice	[52]
	10, 20, 40 μg/mL 25, 50, 100 mg/kg	NADPH oxidase ↓ Angiotensin II ↓ p-JAK2, p-STAT3 ↑	H9C2 cell	STZ-treated C57BL/6 mice	[72]
I/R injury prevention	2.5, 5, 10, 20, 40, 80 μM 5, 10, 20 μM	ER stress ↓ Cell apoptosis ↓ Nrf2, HO-1 ↑ Activation of PI3K/AKT pathway	H9C2 cell, Langendorff-perfused SD rat heart model		[73]
	5, 15 mM	Bax, cytochrome C ↓ Malondialdehyde ↓ Bcl-2 ↑ SOD, GSH-PX ↑	Langendorff-perfused SD rat heart model		[5]

↑: increase; ↓: decrease; TBARS: *thiobarbituric acid reactive substances*; HDL: high-density lipoprotein; MPO: myeloperoxidase; LDL: low-density lipoprotein; HMG-CoA: 3-hydroxy-3-methylglutaryl-coenzyme A; apoB: apolipoprotein B; apoA-I: apolipoprotein A-I; ACE: angiotensin-converting enzyme; ROS/RNS: reactive oxygen and nitrogen species; NADPH: nicotinamide adenine dinucleotide phosphate; L-NAME: N*_ω_*-nitro-*_L_*-arginine methyl ester; COX2: cyclooxygenase-2; NOS: nitric oxide synthase; SHR: spontaneously hypertensive rat; *eNOS*: Endothelial nitric oxide synthase; PI3K: phosphoinositide-3 kinase; cGMP: cyclic guanosine monophosphate; Dox: doxorubicin; ISO: isoproterenol; Nrf2: nuclear factor erythroid 2-related factor 2; HO-1: heme oxygenase-1; iNOS: Inducible nitric oxide synthase; OPN: osteopontin; HMGB1: high mobility group protein B1; CaMKII: calcium/calmodulin-dependent protein kinase II; RIPK3: receptor-interacting serine/threonine-protein kinase 3; IL-6: interleukin 6; JAK: Janus kinase; STAT3: signal transducer and activator of transcription 3; PPARs: peroxisome proliferator activated receptors; PGC-1α: PPARG coactivator 1 alpha; NF-kB: nuclear factor kappa-light-chain-enhancer of activated B cells; HIF-1α: hypoxia-inducible factor-1α; VEGF-α: vascular endothelial growth factor-α; TGF-β: transforming growth factor-β; FGF21: fibroblast growth factor21; AKT: protein kinase B; mTOR: mammalian target of rapamycin; MMP-9: matrix metallopeptidase-9; TIMP1: tissue inhibitor of metalloproteinases-1; MAPKs: mitogen activated protein kinases; TAC: transverse aortic constriction; STZ: streptozotocin; ER: endoplasmic reticulum; Bax: Bcl2-associated X; Bcl-2: B-cell lymphoma-2; SOD: superoxide dismutase; GSH-PX: glutathione peroxidase; SD: standard deviation.

## Data Availability

Data available on request from the authors.
